# Role of autophagy in neurotoxic protein’s clearance following post-ischemic stroke: where we are and what we know?

**DOI:** 10.1186/s13041-025-01201-1

**Published:** 2025-07-08

**Authors:** Sareh Kazmi, Fatemeh Farokhi-Sisakht, Samin Davoody, Gozal Bahlakeh, Fatemeh Abbaszadeh, Reza Rahbarghazi, Aliakbar Shekarchi, Mohammad Karimipour

**Affiliations:** 1 Department of Neuroscience, Faculty of Advanced Medical Sciences, Tabriz University of Medical Sciences, Tabriz, Iran; 2 Neurosciences Research Center (NSRC), Aging Research Institute, Tabriz University of Medical Sciences, Tabriz, Iran; 3 Student Research Committee, Yasuj University of Medical Sciences, Yasuj, Iran; 4 Student Research Committee, School of Medicine, Shahid Beheshti University of Medical Sciences, Tehran, Iran; 5 Department of Anatomical Sciences, Faculty of Medicine, Tabriz University of Medical Sciences, Golgasht Street, Azadi Avenue, Tabriz, 5166614756 Iran; 6 Neurobiology Research Center, Shahid Beheshti University of Medical Sciences, Tehran, Iran; 7 Stem Cell Research Center, Tabriz University of Medical Sciences, Tabriz, Iran; 8 Department of Applied Cell Sciences, Faculty of Advanced Medical Sciences, Tabriz University of Medical Sciences, Tabriz, Iran; 9 Department of Pathology, Faculty of Medicine, Tabriz University of Medical Sciences, Tabriz, Iran

**Keywords:** Protein aggregates, Ischemic stroke, Autophagy, Neuroinflammation, Oxidative stress, Neurodegeneration

## Abstract

**Background:**

The role of autophagy following stroke and its underlying cascades have not yet been investigated in detail. The ischemic brain is characterized by complex pathophysiological mechanisms, including increased excitotoxicity, oxidative stress, inflammatory responses, intrinsic and extrinsic apoptotic pathways, blood-brain barrier (BBB) integrity, neurotoxic proteins, and neurodegeneration. By engaging multiple molecular pathways, autophagy plays both protective and detrimental roles in ischemic stroke. **Main text**: This review explores the state-of-the-art regarding autophagy’s role in neurotoxic protein clearance, neuroinflammation, oxidative stress, BBB, and neural tissue regeneration during and after ischemic stroke. Additionally, neuroinflammation is modulated by autophagy such that the inflammasomes and proinflammatory complexes that cause post-ischemic neuroinflammation are degraded. However, autophagy can be dysregulated, resulting in chronic neuro-inflammation. Moreover to counteract the excessive oxidative stress, autophagy is triggered mainly through the PINK1/Parkin pathway. In contrast, over-activated autophagy may cause neuronal damage and cell death. Autophagy maintains BBB integrity by restoring tight junction proteins. However, if dysregulated, the infiltration of inflammatory neurotoxic substances can exacerbate ischemic injury, highlighting the need for balanced regulation of autophagy. As the central nervous system (CNS) has limited regenerative capability, neural stem and progenitor cells are activated to promote neurogenesis following stroke. Autophagy can also enhance those regenerative processes. **Conclusions** Modulating autophagy offers potential therapeutic strategies in stroke patients by enhancing the protective effects of autophagy while minimizing its harmful consequences.

**Graphical abstract:**

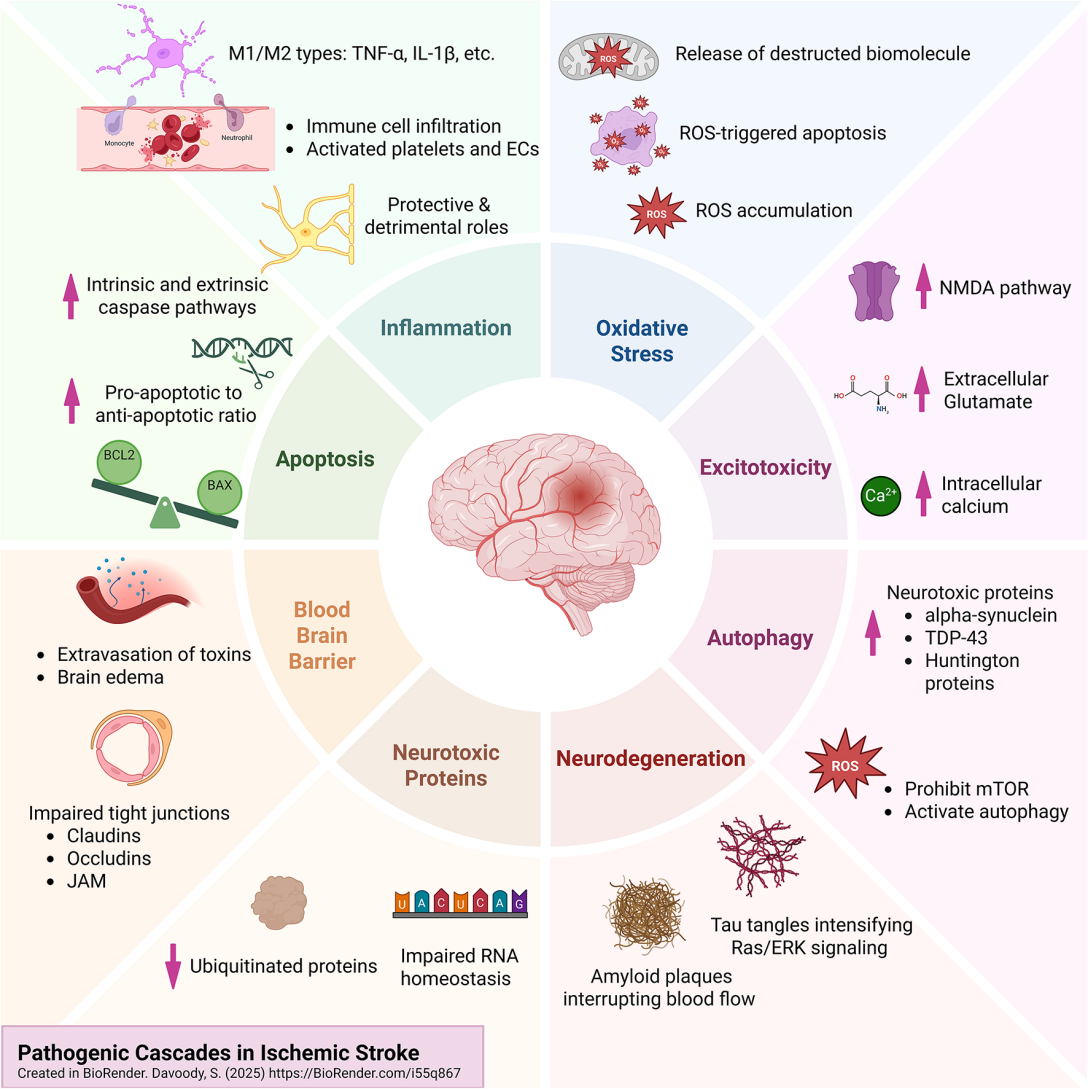

## Introduction

Stroke is the second leading cause of human death globally, and the main cause of disability in developing countries [1]. Stroke is estimated to affect 17 million people annually, with a mortality of 5.5 million individuals [2, 3]. In terms of regional incidence, stroke is common in East Asia and Eastern European countries, while central Latin America has the lowest incidence rates [4]. Based on previous data, young females and aged males are at risk of stroke. Despite these features, the mortality rate is higher in males compared to females [4, 5]. From the clinical manifestation, stroke can contribute to physical disability and cognitive deficits. Despite the existence of numerous studies on the analysis of physical disorders in stroke patients, cognitive impairments and relevant outcomes have mostly been neglected [6]. Of note, approximately one-third of stroke survivors suffer from cognitive dysfunction with prominent effects on quality of life and socioeconomic burden [7, 8]. With the occurrence of stroke in patients, the rate of cognitive impairment is 5-to 8-fold higher than that in their healthy counterparts [9]. Stroke is generally classified into ischemic and hemorrhagic. Ischemic stroke is the main type of stroke (nearly 87%) and occurs because of stenosis or occlusion of cerebral arteries. Thrombus formation, the existence of emboli, and atherosclerosis predispose patients to ischemic stroke [10, 11]. With a lower prevalence rate, hemorrhagic stroke occurs due to bleeding or rupture of vessels and accumulation of blood in the brain parenchyma (intracerebral hemorrhage) or subarachnoid space (subarachnoid hemorrhage) [12].

### Ischemic stroke

The human brain is a highly energy-intensive tissue that constitutes about 2% of total body weight. Approximately 20% of the total metabolic rate belongs to neuronal cells within the brain parenchyma [13]. Therefore, suitable blood perfusion should be provided for proper neurological activities in which the cerebral blood flow (CBF) rate is around 50 ml per 100 g per minute in a healthy adult person [14]. When the CBF rate reaches below 40% of normal values, the brain tissue is sensitive to ischemic conditions [15]. In the context of pathological outcomes, ischemic brain tissue consists of an ischemic core and a penumbra [16]. In the ischemic core, the CBF rate is less than 10 ml/100 g/minute and these features result in bulk neuronal cell death enclosed by the blocked vessel. Notably, ischemic neurons cannot restore their physiological activities even after the establishment of blood flow [17, 18]. Penumbra is about half of the ischemic lesion volume with a lower CBF rate (~ 35 ml/100 g/minute). Compared to neurons located in the ischemic core, penumbra region neurons are more resistant to ischemia because they are relatively juxtaposed with collateral blood flow [17, 18]. In response to ischemic conditions, neurons and glial cells exhibit morphological alterations. For instance, severe hydropic degeneration leads to prominent swelling and loss of soma and axons in neurons in the ischemic core. Along with these changes, glial cells are also swollen. In the penumbra, massive degeneration of endoplasmic ribosomes and Nissl bodies is evident inside the affected neurons. These features contribute to the activation of microglia and polarization into M1 cells, in which swollen ameboid-like cells can be detected with the ability to produce diverse pro-inflammatory cytokines [19]. Similar to microglia, astrocytes undergo morphological changes and abundant alterations in their transcriptomic profiles [20]. According to the time of the process occurring at the affected sites, ischemia is classified into three different stages: the acute phase (lasting for hours), the sub-acute phase (lasts from hours to days), and the chronic phase (lasts from days to months) [21]. In the acute stage, ischemic injury happens due to abrupt reduction and/or interruption of CBF and subsequent reperfusion. Along with these changes, a cascade of molecular and cellular events is initiated. Because of ischemic/reperfusion injury, neuronal cell excitotoxicity, oxidative stress, and inflammation lead to bulk cell death in ischemic injury [22]. With the progression of vasogenic edema, the acute phase begins [23]. It has been thought that delayed regeneration or inefficiency of repair mechanisms are responsible for irreversible chronic pathologies. Oxidative stress and immune cell activation are integral to induction of the chronic phase [22].

### Cellular and molecular mechanisms in ischemic stroke

#### Excitotoxicity

The term “excitotoxicity” refers to the injury of neurons and glial cells induced by overactivation of glutamate- N-methyl-D-aspartate (NMDA) pathway [24]. Several studies have indicated that glutamate is the main excitatory neurotransmitter in the mammalian central nervous system (CNS). This neurotransmitter plays a crucial role in several brain activities such as neuronal development, synaptic plasticity, and cognition [25, 26]. Maximum levels of glutamate exist in nerve endings. Specifically, these values can reach 100 mM and 10 mM in the synaptic vesicles and presynaptic cytoplasm, respectively. Within the extracellular space, the glutamate levels are low (~ 1 µM) [27]. It is postulated that trivial levels of extracellular space glutamate can be taken up by astrocytes and neurons [28]. Following the occurrence of ischemic conditions, glutamate homeostasis is disrupted due to energetic stress (ATP↓) and loss of energy-dependent pump activity [29]. In hypoxic and ischemic neurons, the intracellular ATP content falls below critical levels; thus, the functions of membrane-bound pumps such as Na^+^/K^+^-ATPase, Na^+^/Ca^2+^-ATPase, and Ca^2+^-ATPase are blunted [30]. Upon an increase in intracellular Ca^2+^, the release of glutamate is promoted into the synaptic space [31]. The concomitant reduction in glutamate uptake by glial cells (astrocytes) exacerbates the accumulation of glutamate in synaptic space [32]. The attachment of glutamate to NMDA receptors in the postsynaptic membrane increases excessive intracellular Ca^2+^ accumulation and activation of the cell death signaling cascade [33].

#### Oxidative stress

Neurons exhibit eminent metabolic activity with high oxygen consumption, making them sensitive to oxidative stress [34]. Oxidative stress occurs due to dysfunction of the antioxidative system, resulting in the generation and accumulation of toxic reactive oxygen species (ROS) and the destruction of biomolecules [35]. Oxidative stress has been described in neurons exposed to ischemic condition, and the re-establishment of brain-blood flow (BBF) after ischemia predisposes neurons to oxidative stress [36]. The subcellular units, such as mitochondrial oxidative phosphorylation system with multiprotein complexes, such as electron transfer chain, and nicotinamide adenine dinucleotide phosphate (NADPH) oxidases (NOX) family, are the main sources of ROS production [37]. Under ischemic conditions, the normal structure and function of mitochondria are lost, and numerous small fragments are produced inside injured neurons [38]. Following cerebral reperfusion, considerable amounts of ROS are generated, coinciding with the loss of antioxidant system activity and disturbance of the mitochondrial antioxidant-oxidant system. In the presence of excessive ROS, the activity of the electron transport system is inhibited in the inner mitochondrial membrane, which per se reduces the production of ATP. Loss of selective permeability in the mitochondrial membrane increases the release of cytochrome C and activation of apoptotic death [18, 39]. NOXs are membrane-bound protein complexes that utilize NADPH as a substrate and transfer electrons from the plasma membrane to oxygen molecules to produce ROS [40]. In cerebral ischemia, distinct NOX isoforms (NOX2 and 4) are expressed to mediate ROS-triggered apoptosis [41].

#### Inflammation

**Fig. 1:**
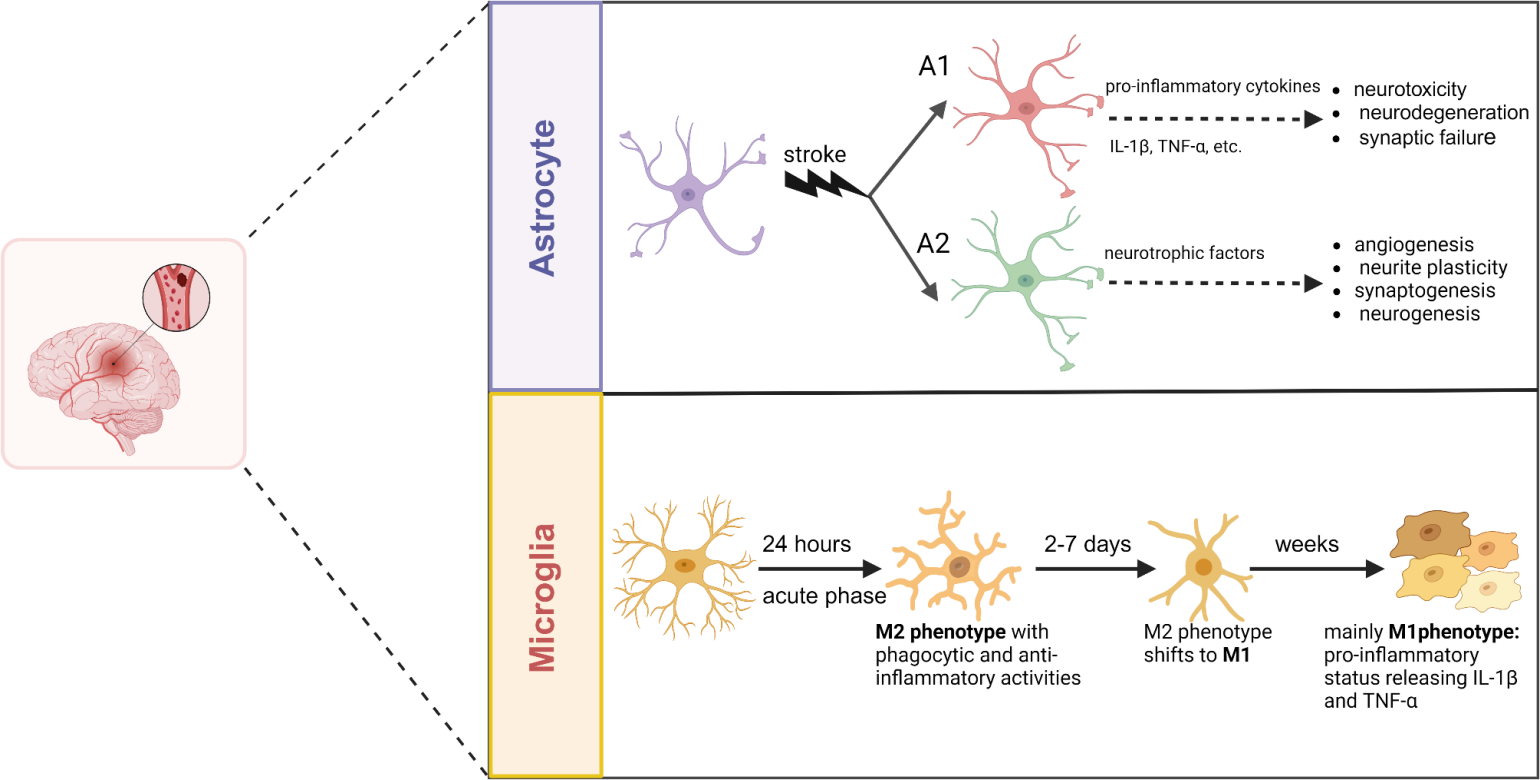
The phenotypic changes and functions of astrocytes and microglia in response to stroke. Upon stroke, astrocytes differentiate into mainly A1 and A2 phenotypes. A1 astrocytes (red) release pro-inflammatory cytokines (e.g., IL-1β and TNF-α), resulting in the exacerbation of the ischemia-related injuries. A2 astrocytes (green) mainly protect neural cells by secreting neurotrophic factors. Soon after brain injury, microglia leave the resting state, and the soma gets enlarged and branches get thickened. The exposure of microglia to the damage sites results in an M2 phenotype (phagocytic and anti-inflammatory) 24 h post-stroke, which transitions to the M1 phenotype (pro-inflammatory) over the following weeks, releasing cytokines such as IL-1β and TNF-α. Created in BioRender. Davoody, S. (2025) https://BioRender.com/s84i284

It is well known that microglia and astrocyte-derived mediators are responsible for inflammatory responses in the nervous system [42]. Upon the occurrence of ischemic conditions, brain microglia are activated during the early stages (Fig. 1). It is estimated that these cells are provoked at an early time-point between 3.5 and 12 h after ischemia. The number of microglia reaches maximum levels at ischemic sites after seven days [43, 44]. Under physiological conditions, microglia are in a resting state and have a small cell body with ramified processes and branched projections [43]. Soon after brain tissue injury, microglia are activated and their morphological features shift. The ramified branches begin thickening and shortening and the soma becomes enlarged. Notably, the migrating microglia toward the ischemic site exhibit an M2 phenotype with prominent phagocytic and anti-inflammatory activities. The exposure of microglia to damage signals accelerates the phenotypic shift from M2 to M1. M1 microglia exhibit pro-inflammatory status with the potential to produce several cytokines including interleukin-1β (IL-1β) and tumor necrosis factor-α (TNF-α) [45, 46]. The release of such cytokines can activate juxtaposed endothelial cells (ECs), platelets, and lymphocytes. Under such conditions, the expression of adhesion molecules such as selectins (ICAM-1, VCAM-1, etc.) is induced, resulting in immune cell infiltration (neutrophils and monocytes) and loss of blood-brain barrier (BBB) integrity [47]. Similar to microglia, astrocytes belong to the brain defense system. The release of damage signals by neurons and activated glial cells triggers local astrocytes within minutes of ischemic stress. The reactive astrocytes undergo morphological changes such as hypertrophy, and produce a large number of intermediate filamentous proteins (glial fibrillary acidic protein, vimentin, and nestin) [48, 49]. Both protective and detrimental roles of astrocytes during cerebral injury have been proposed [19]. In support of this notion, the reactive astrocytes (A1 type) can release pro-inflammatory cytokines (IL-1β, TNF-α, etc.), which in turn exacerbate the ischemia-related injuries. In contrast, the A2-type astrocytes can protect the neuronal cells by engaging multiple mechanisms, including releasing neurotrophic factors, inducing angiogenesis, neurite plasticity, synaptogenesis, and neurogenesis [1]. Pathological proliferation of astrocytes is called astrogliosis. This phenomenon can form a thick layer composed of filament proteins around the ischemic core, which is called a glial scar [50]. Glial scar prohibits axonal sprouting and neuroregenerative processes. However, this layer can function as a protective barrier that separates damaged tissue from viable regions and limits the diffusion of cytotoxic molecules to viable neural tissues [50, 51].

#### Apoptosis

Apoptosis is an important portion of cells in the ischemic penumbra [52]. This phenomenon coincides with the generation of apoptotic bodies, condensed chromatin, and shrinkage of whole cell mass and nucleus. Activation of intrinsic endonucleases cleaves nuclear deoxyribonucleic acid and yields 180–220 bp fragments [53]. Both the intrinsic (caspase 9-dependent) and extrinsic (caspase 8-dependent) pathways are initiated in apoptotic cells during cerebral ischemia. In both pathways, caspases 8 and 9 activate the downstream caspases 3 [54]. In the intrinsic pathway, cerebral ischemia disrupts the normal electrochemical gradient across the mitochondrial membrane [53]. It has been indicated that B-cell lymphoma 2 (Bcl-2) family proteins are important effectors in mitochondria-related apoptosis. The proteins of this family are in close contact with the mitochondrial outer membrane, and have anti-apoptotic (Bcl-2) and pro-apoptotic Bcl-2-associated X protein (Bax) activities. The increase in the pro-apoptotic to anti-apoptotic protein ratio triggers molecular cascades associated with apoptotic cell death. In response to ischemic conditions, the reduced expression of Bcl-2 coincides with the upregulation of pro-apoptotic effectors, such as Bax [55, 56]. As a result, loss of mitochondrial membrane permeability contributes to the release of the pro-apoptotic factor cytochrome C into the cytosol [57]. The close interaction of cytochrome C with other effectors, such as Apaf1, triggers apoptosome complex formation. In the next step, inactive pro-caspase 9 is recruited, leading to the activation of executive caspase 3 [58, 59]. Compared with the intrinsic apoptotic pathway, the extrinsic apoptotic pathway depends on the activation of membrane-bound death receptors belonging to the superfamily of tumor necrosis factor receptors. The carboxyl terminus of these receptors binds to pro-caspase 8 and forms the death signaling complex. Upon the attachment of cognate ligands, caspase 8 is activated, which in turn stimulates caspase 3 using proteolytic cleavage [60]. Some studies have indicated that the active caspase 8 can indirectly promote mitochondria-related apoptotic pathways via the proteolysis of Bid and generation of tBid [61].

#### Blood brain barrier

BBB is a natural barrier that supports regular operation of the CNS. The BBB acts as a highly selective barrier to isolate blood and cerebrospinal fluid (CSF) [62]. Ultrastructural images have revealed that BBB is a semipermeable barrier with the potential to provide a microenvironment suitable for normal neuronal activity in the CNS. This barrier regulates the entrance of nutrients and the elimination of harmful byproducts [63]. BBB integrity is maintained by ECs, tight junctions (TJs), pericytes, astrocytes, and the extracellular basement membrane (BM) [64]. ECs furnish the luminal surface of cerebral blood vessels [65]. The structure and connection of ECs within the brain parenchyma differs from those of vascular ECs located in other tissues. Brain ECs are tightly connected without fenestrations. These cells lack pinocytic activity and have several transport mechanisms that control the passage of essential molecules into the brain [66]. TJs are found between juxtaposed ECs at the apical surface. It has been thought that TJs prevent the crossing of circulating hydrophilic compounds (180 Da < X) from the bloodside to thr brain tissue [67]. The three types of TJs are claudins, occludins, and junctional adhesion molecules (JAM). These transmembrane proteins are connected to scaffold effectors, i.e., zonula occludens (ZO), via their cytoplasmic domains [68, 69]. Among these molecules, claudins are actively involved in the formation of a paracellular barrier between the ECs and other epithelial cells. The molecular distribution of these molecules depends on tissue type [70]. Claudin-5 is an abundant TJ protein at the BBB interface. Of note, it has been indicated that the expression of this protein is about 1000-fold brain ECs compared to other claudins [71, 72]. In addition, claudin-5 actively participates in the entry of small molecules through the BBB and contributes to enhanced endothelial layer resistance and selective BBB permeability [73].

Similar to claudins, occludins function as TJs to maintain BBB interface integrity [74]. Occludins are dimeric and regulate the paracellular permeability of ECs [75]. The last member of the TJs family is JAMs, which belongs to the immunoglobulin superfamily and participates in reciprocal cell-to-cell or cell-to-ECM interactions. Among different JAMs, JAM-A directly affects TJ functions [76]. As mentioned above, ZOs are cytoskeleton linkers with the potential to regulate cytoskeleton mobility by direct physical contact with F-actin [77].

At the abluminal surface, ECs are wrapped by cytoplasmic projections of pericytes [78]. Histological analyses have indicated the presence of BM between ECs and pericytes. Pericytes and ECs are juxtaposed via connexins and N-cadherin [79]. Within the brain tissue, pericytes control the CBF rate via the regulation of capillary diameter [80]. Scientific evidence points to the fact that the phagocytic activity of pericytes can help the BBB interface eliminate toxic/harmful compounds and their entrance into the brain [81]. Certain signaling pathways, such as angiopoietin-1/Tie-2, platelet-derived growth factor (PDGF), and transforming growth factor (TGF-β), are involved in the stabilization of the BBB structure via the maintenance of EC-to-pericyte communication [82].

Astrocytes contribute to maintaining the integrity of BBB structure as well. These cells are closely juxtaposed to the vascular structure by a flattened process called endfeets. It has been estimated that nearly 99% of the brain microstructure is wrapped by astrocyte endfeets [83, 84]. To be specific, astrocyte endfeets can connect the brain vessels and neurons and help regulate the water and ions, and interchange signaling molecules [85, 86]. The release of certain signaling molecules such as Wnt and Shh by endfeets increases the integrity of BBB interface.

#### BBB disintegrity

Most nervous system disorders contribute to BBB dysfunction and the loss of integrity. Because of the BBB integrity removal, several harmful substances and xenobiotics can enter the brain parenchyma, leading to cognitive disorders and motor deterioration [87]. In response to different pathological conditions, such as ischemic stroke, TJ proteins are not functional, and thus, BBB integrity is abolished [88]. Hypoxic ECs are shrunken because of the translocation of TJs from the membrane into the cytosol [89]. It is thought that reperfusion can exacerbate the loss of BBB integrity and promote the development of cerebral edema [90]. Accumulated ROS can increase BBB dysfunction via different mechanisms, such as TJ protein function, cytoskeletal remodeling, activation of the kinin system, immune cell recruitment, activation of microglia, and glutamate toxicity [91]. Following cerebral ischemia, the production of advanced glycation endproducts (AGEs) upregulates NF-κB inside microglia and induces a pro-inflammatory phenotype via the production of TNF-α and IL-1β [92]. These cytokines induce apoptotic changes and increase matrix metalloproteinases (MMPs) in neurons and other glial cells, leading to loss of BBB integrity. Further activation of ECs provokes immune cells and intensifies detrimental effects under ischemic conditions [93–96]. Active MMPs, i.e. MMP-2 and − 9, decompose the BM of BBB, and their influences have been demonstrated in several pathological conditions such as Alzheimer’s disease (AD), multiple sclerosis (MS), epilepsy, Parkinson’s disease (PD), stroke, and brain cancers [97]. The lack of an integrated BBB structure leads to several plasma proteins leaking into the brain space and subsequent water entry, resulting in brain edema [98]. Among several MMP types, MMP-2, and − 9 digest type-IV collagen, laminin, and fibronectin in the brain microvascular structure [99]. The contents of MMPs are high during hours to days after stroke, which can distort the function of occludin, claudin-5, and ZO-1 [95, 100]. Along with MMP-2, and − 9, MMP-12 degrades elastin, type IV collagen, laminin, fibronectin, and vitronectin [101]. These features increase the infiltration of immune cells into the brain parenchyma [96, 102, 103]. The recruited immune cells, such as neutrophils, can release several cytokines, such as MMP-9, and oxidants, leading to the worsening of inflammatory conditions [103, 104]. In animal models of BBB degeneration, the loss of BBB integrity occurs in two distinct phases. With the initiation of permeability within the BBB structure, a refractory reaction occurs, and the permeability returns to baseline values. This phase is followed by a secondary reaction with an enhanced permeability [105, 106]. However, other studies have not shown a biphasic opening of the BBB structure. It is postulated that the loss of BBB integrity occurs during the 24 h after ischemic conditions and remains stable for up to 7 days [107, 108].

#### Aggregation of neurotoxic proteins

Under physiological conditions, proteins possess specific structures in the aqueous phases to exert appropriate biological functions. The loss of a normal 3D structure can predispose the aggregation of diverse protein factors within the brain parenchyma and the occurrence of neurodegenerative disorders such as AD, amyotrophic lateral sclerosis (ALS), and PD. The loss of normal molecular structure increases the retention time and aggregation, resulting in neurotoxicity [109, 110]. Similar to several neurodegenerative diseases, aggregated proteins are deposited following ischemic stroke, leading to neurotoxicity and neuronal cell death [111, 112]. The prominent changes in gene expression and increase in misfolded proteins are common features between ischemia and neurodegeneration [113]. However, mechanisms underlying protein aggregation under ischemic conditions remain uninvestigated. Among the several intracellular mechanisms involved in protein homeostasis, the ubiquitin-proteasome system (UPS) plays a critical role in the regulation of protein functions [114]. Abnormal reduction in UPS function can predispose the intracellular accumulation of ubiquitinated proteins, resulting in neuronal cell death and neurodegenerative disorders [115]. The increase in ubiquitinated proteins following cerebral ischemia can lead to loss of protein degradation and proteotoxicity [116, 117]. It is thought that ubiquitinated proteins can be assembled to form large-sized aggregates that sensitize neurons to facilitate atresia [118–120]. For instance, two RNA-binding proteins, TAR DNA-binding protein 43 kDa (TDP-43) and translocated in liposarcoma (FUS/TLS), are abundant in ALS, frontotemporal dementia (FTD), and some AD cases [121]. It has been suggested that aggregated proteins can alter the physiology of RNA and metabolism, as described in ischemia [122]. Elevation of TDP-43 levels following ischemic stroke can intensify mitochondrial dysfunction. In response to the accumulation of TDP-43, compensatory gliosis occurs [123]. Likewise, FUS can also alter RNA homeostasis and metabolism under several pathological conditions, leading to reduced cell survival rates and progression of neurodegeneration. In ischemic neurons with abnormal FUS content, reactive astrocytes induce neuronal cell death via stimulation of autophagy [124]. Studies have also confirmed that ischemic reperfusion injury may mimic AD-like gene expression and misfolded protein accumulation [125–127].

#### Ischemic stroke and the development of neurodegeneration

Several studies have shown the possibility of brain neurodegeneration post-ischemia, with impaired cognition (~ 70%) and dementia [128, 129]. However, the mechanisms by which post-stroke neurodegeneration occurs required further investigation. In ischemic stroke survivors with disabilities and deficits, changes in the levels of amyloid precursor protein (APP) and tau protein, and expression of β-secretase, presenilin 1, and presenilin 2 are evident [130–132]. Both vasoconstriction and amyloid plaques can interrupt blood flow within the CNS and cause massive neuronal cell death [133–135]. Along with these changes, the accumulated tau protein stimulates neuroinflammation via inhibition of the transportation of APP vesicles, neurofilaments, and even organelles, and the production of ROS [136–139]. Excessive ROS production after ischemia stimulates several signaling cascades associated with the phosphorylation of tau protein and neurofibrillary tangle (NFT) formation [140]. Tau protein intensified excitotoxic brain lesions through Ras/ERK signaling downstream of NMDRs in stroke models [141]. Besides, the abnormal activity of other signaling pathways, such as Ca^2+^, acetylcholine, and metal ions, may be related to ischemia-related neurodegeneration [142, 143]. The complete loss or partial activity of intracellular scavenging mechanisms, such as autophagy and mitophagy, is also integral to the occurrence of neurodegeneration in neural cells [144, 145].

#### Autophagy

**Fig. 2:**
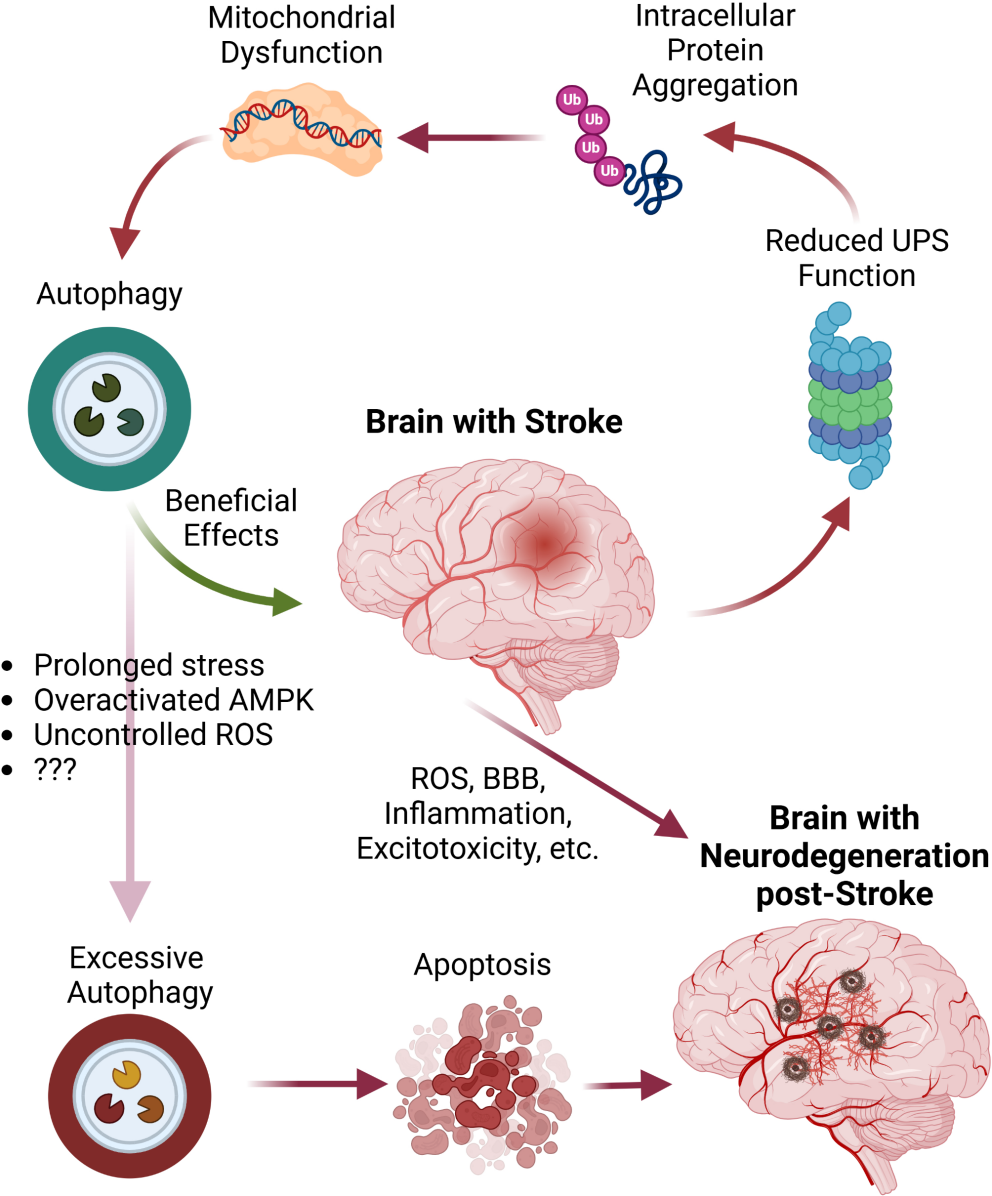
Autophagy and its dual role in post-stroke neurodegeneration. Following stroke, autophagy facilitates the clearance of damaged cellular components, exerting beneficial effects on neurons. However, sustained pathological conditions can lead to excessive autophagy. Dysregulated autophagy results in apoptotic cell death contributing to neurodegeneration. Created in BioRender. Davoody, S. (2025) https://BioRender.com/tn2cxjc

Autophagy is a self-eating mechanism that is involved in the maintenance of cell homeostasis. This phenomenon is an intricate and selective process for the elimination of misfolded proteins, and is tightly regulated by several autophagy-related genes (ATGs). Three forms of autophagy exist in eukaryotic systems: microautophagy, chaperone-mediated autophagy (CMA), and macroautophagy [146]. The term microautophagy refers to the direct elimination of cytosolic materials by lysosomal degradation. The targeted biomolecules and materials are directed toward lysosomes, and digestion is initiated after internalization into the lysosome lumen. In CMA, proteins with KFERQ-like motifs are recognized and guided toward lysosomes. The most dominant form of autophagy is macroautophagy, herein known as autophagy, which is activated to eliminate the exhaust materials using the specialized double-membranes, namely autophagosomes. Further fusion of autophagosomes with lysosomes leads to the formation of autophagolysosomes. Using hydrolases, the enclosed materials are digested and the breakdown components are recycled for biosynthesis and/or released into the ECM [147]. In healthy cells, adaptive autophagy is activated in response to external stimuli to preserve cellular homeostasis. However, under pathological conditions, the activation of excessive autophagy response can lead to restoration of cell function and/or cell death. Conditions such as hypoxia, starvation, oxidative stress, microbial pathogens, organelle damage, and the accumulation of aggregated proteins such as Aβ and tau protein can stimulate autophagy [148]. The reduction of CBF in brain ischemia predisposes to profound changes in the metabolic profile due to the lack of O_2_ and hypoglycemia. The reduction of ATP leads to activation of autophagy, prominent cytoskeletal remodeling, and morphological changes. Under severe conditions, an excessive autophagic response activates other cell death mechanisms such as apoptosis [149]. In terms of the distinct molecular mechanisms provoked for autophagy, cells use certain autophagic responses. For instance, the term pexophagy refers to the elimination process of aged or dysfunctional peroxisomes. Besides, mitophagy is activated to eliminate the injured or accumulated mitochondrial particles. Xenophagy is the clearance of intracellular xenobiotics such as microbes or other non-pathogenic particles. Aggrephagy is a compensatory response to digest misfolded proteins that occur during neurodegenerative disorders [150] (Fig. 2).

#### Autophagy and neurotoxic proteins

Neurotoxic proteins are produced from different sources under pathological conditions and predispose neurons to atresia and abnormal brain function. Bacterial toxins such as botulin, tetanus, anthrax, alcohol (ethanol), viral particles, fungi, plants, and protozoa, are the most common causes of toxicity within the CNS. In response to these conditions, the overproduction or accumulation of neuronal cell-derived proteins increases. With the progression of such conditions, aggregation is evident due to protein misfolding. Genetic traits, pathological conditions, and aging can facilitate the formation of protein aggregates inside the neurons or in the ECM space. For instance, Aβ oligomers, tau protein, RNA-binding proteins (TDP-43, TIA-1, and FUS), alpha-synuclein, and Huntington (Htt) proteins are the most common aggregates within the brain parenchyma. The presence of such proteins influences the metabolic status, inter-cell communication, and paracrine activity of host neurons. With the continuation of protein aggregates, inflammatory response, oxidative stress, and thereby neuronal cells occur in patients with AD, PD, ALS, and Huntington’s disease [151]. To eliminate neurotoxins, endogenous mechanisms, such as the glymphatic system, BBB, UPS, and autophagy, are activated. The BBB and glymphatic system are the dominant scavenging mechanisms to eliminate protein aggregates from the neuronal ECM, interstitial fluid (ISF), and CSF. Receptors, such as p-glycoproteins and low-density lipoprotein receptor-related protein 1 (LRP1), are important factors for clearing aggregates. Glial cell phagocytosis by astrocytes and microglia can help eliminate neurotoxins in the CNS [152, 153]. Inside the neurons, two main systems, the UPS and autophagy, can help host cells to clear aggregated proteins. The UPS system targets specific proteins for degradation by the ubiquitin molecules inside proteasomes. While in autophagy, target molecules are sequestrated by autophagosomes and lysosomal degradation [152, 154]. Thus, these mechanisms are essential for maintaining the physiology of neurons and alleviating neurodegenerative diseases.

#### Autophagy and neuroinflammation

Neuroinflammation is defined as the emergence of inflammation in the CNS in response to diverse stimuli that can lead to CNS injury. Although acute inflammation can foster healing of CNS, chronic inflammation can contribute to aberrant remodeling, loss of physiological function, and neurological disorders. It has been thought that cytokines such as TNF-α, IL-6, and − 18 along with chemokines (CCL2, CCL5, and CXCL3), NO, and ROS, are produced abundantly during neuroinflammation [155]. These cytokines are mainly released by resident glial cells such as microglia [156]. The continuity in production and release of these cytokines leads to neuronal cell damage via excitotoxicity, apoptosis, and necrosis. In the presence of pro-inflammatory cytokines, the BBB loses its integrity and increases immune cell infiltration (i.e., neutrophils), leading to the worsening of CNS diseases. Inflamed microglia cannot eliminate misfolded proteins, leading to accumulation of α-synuclein, Aβ, and tau protein. These features show that anti-inflammatory agents targeting TLRs, NF-κB, and NLRP3 inflammasomes can be used as therapeutics for the alleviation of neurological diseases. Emerging data have indicated a critical role of autophagy in CNS pathologies [157]. Both the inhibitory and stimulatory effects of autophagy have been detected in terms of neuroinflammatory responses. However, abnormal autophagic activity can contribute to the aggregation of certain components of neurons. Even though under such conditions, the possibility of damage signals is also increased, leading to enhanced pro-inflammatory response. Notably, autophagy can help immune cells in antigen presentation along with major histocompatibility complex (MHC) molecules. These features help immune cells recognize the immunogenic antigens appropriately and regulate the immune system response [158]. Autophagy is also effective for the production and release of cytokines. The inhibitory and stimulatory capacities are associated with certain pathological conditions and durations. In conditions that coincide with the production of cytokines by autophagic response, immune cells can effectively act against the pathogens. Autophagy can control the immune cell response and overactivity, which are critical for the prevention of uncontrolled tissue damage, inflammation, and maintenance of cell homeostasis [159]. For example, the close interaction between NLRP3 inflammasomes and autophagy machinery occurs at different levels. In general, autophagy can sequester NLRP3 inflammasomes and guide lysosomal degradation to further suppress immune cell activation. In this regard, elimination of damaged mitochondria via mitophagy, the main source of NLRP3 inflammasomes, can prevent subsequent immune cell reactions [160, 161]. In another pathway, autophagy modulates NLRP3 inflammasomes by regulating inflammasome-associated cytokines such as IL-1β and − 18. The degradation of pro-IL-1β and pro-IL-18 can tightly regulate their direct access to inflammasomes and their release from the host cells. On the other hand, active NLRP3 inflammasomes induce autophagy machinery, leading to the elimination of intracellular pathogens, aggregated proteins, and damaged organelles. The last mechanism, also known as inflammasome-mediated autophagy, helps the host neurons maintain homeostasis and inflammation [160–162]. These features indicate that the control of neuroinflammation via modulation of autophagy response is a *de novo* therapeutic tool for different neurodegenerative diseases [163]. For example, rapamycin, an mTOR inhibitor and autophagy inducer, can improve cognitive function in several animal models of neurodegenerative diseases. However, mTOR inhibitors do not apply in the clinical setting because of their immunosuppressive effects [164–166]. Other autophagy modulators such as histone deacetylase (HDAC) inhibitors stimulate ATGs with the potential to reduce neuroinflammation and increase neuronal cell survival during neurodegenerative diseases in animal models [167]. Similarly, protein kinase C (PKC), AMPK, and SIRT1 activators can also influence neuroinflammation, with promising outcomes in preclinical investigations [168]. Taken together, there is a close interplay between autophagy and neuroinflammation, and regulation of autophagy is thought to be a novel therapeutic tool for different neurological diseases.

#### Autophagy and oxidative stress

**Fig. 3:**
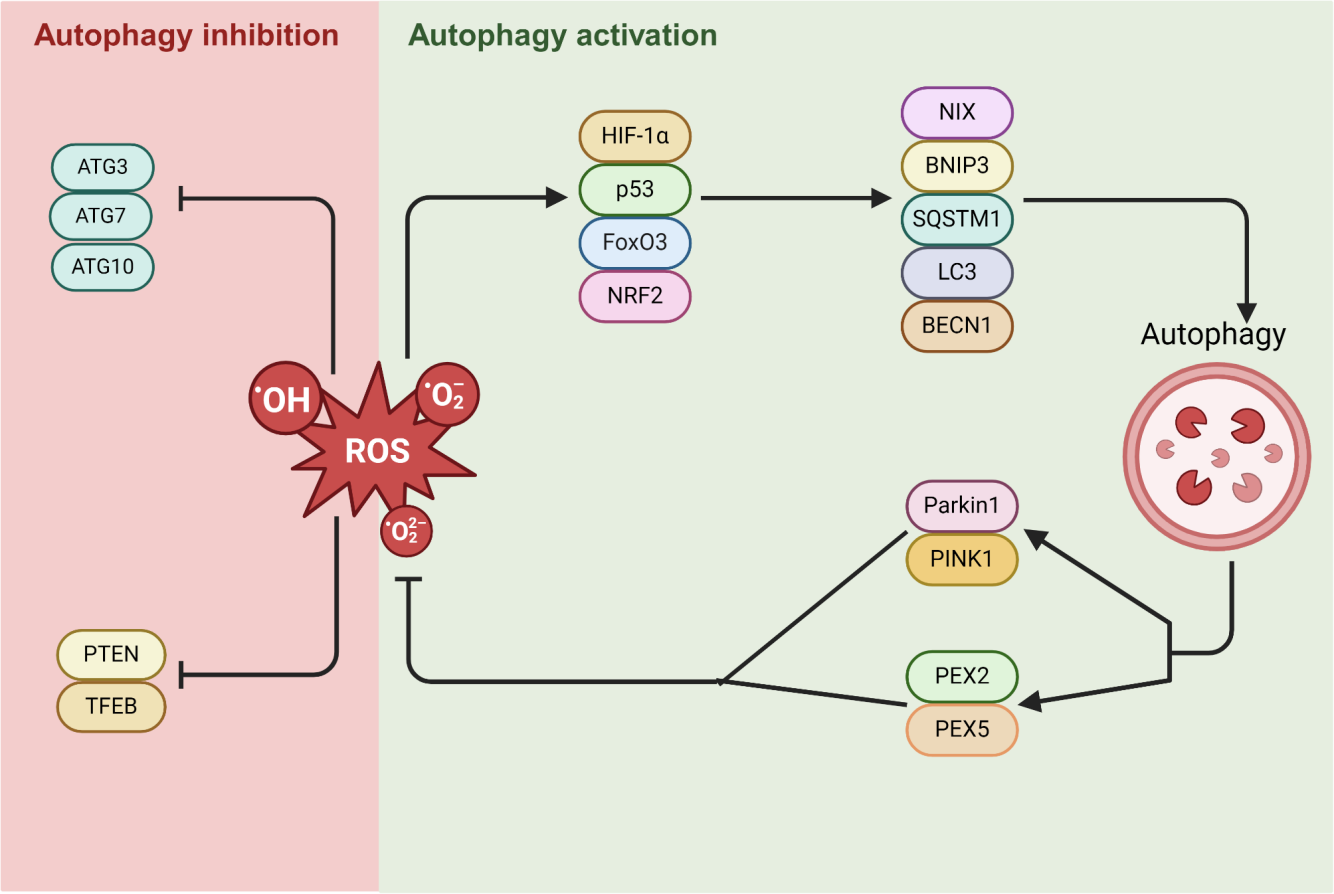
The reciprocal relationship between autophagy and oxidative stress. ROS activity can enhance mTOR signaling by upregulating HIF-1α, NRF2, p53, and FoxO3. These factors in turn activate BECN1, LC3, SQSTM1, BNIP3, and NIX, thereby stimulating autophagy. On the other hand, autophagy activation itself triggers the PINK1/Parkin pathway and engages PEX2 and PEX5 to limit ROS production by removing damaged mitochondria and preventing further mitochondrial damage and ROS release. On the contrary, certain autophagy-related genes (ATGs) are sensitive to ROS and are inhibited by ROS production. Created in BioRender. Davoody, S. (2025) https://BioRender.com/s64u646

ROS, such as O2^•−^, H_2_O_2_, and OH,^·^ are produced during metabolic processes. Excessive ROS production and accumulation due to abnormal antioxidant activity are thought to be predisposed to oxidative stress. Free radicals can invade different biomolecules such as lipids, proteins, and DNA, leading to the disruption of normal cellular functions [169]. Based on emerging data, there is a reciprocal relationship between autophagy and oxidative stress (Fig. 2). ROS can inhibit or stimulate the autophagy machinery. For example, ROS can increase the expression of HIF-1α, NRF2, p53, and FoxO3, which in turn activates BECN1, LC3, SQSTM1, BNIP3, and NIX to maintain redox homeostasis [170]. It is also possible that ROS can inhibit mTOR activity and activate the autophagy machinery [171]. In contrast, some ATGs, ATG7 and 10, are sensitive to ROS, and their inhibition inhibits TFEB and PTEN. Inhibition of PTEN leads to PI3K-AKT-mTORC1 pathway blockade and autophagy suppression. Besides, TFEB can control the expression of effects required for the formation of autophagosomes and lysosome formation. The dual activity of ROS on autophagy is an effective strategy for controlling the balance between pro- and anti-autophagy mechanisms [172]. On the other hand, autophagy can influence the intracellular ROS content. The removal of damaged organelles, subcellular compartments, and protein aggregates diminishes the production of ROS, resulting in the maintenance of redox balance [173]. Free radicals, such as O2^•−^ and H_2_O_2_ generated by mitochondria, can activate autophagy [174]. The damaged mitochondria are removed using PINK1/Parkin and Bnip3/Nix pathways. The PINK1/Parkin pathway induces PTEN-induced putative kinase 1 (PINK1) and E3 ubiquitin ligase Parkin. During mitochondrial damage, PINK1 accumulates on the outer mitochondrial membrane and recruits Parkin. Parkin has the potential to ubiquitinate proteins on the surface of damaged mitochondria, marking them for autophagosome degradation [175, 176]. The Bnip3/Nix-mediated pathway is activated in response to hypoxic conditions, attaches closely to the mitochondrial membrane, and recalls autophagosomes [175]. Proteins such as p38MAPK, ERK, and JNK have also been found to be involved in the induction of autophagy in the presence of abundant ROS [177] (Fig. 3). Generally, ROS stimulates autophagy to eliminate/repair injured organelles and components. Under conditions of excessive ROS levels, the inhibition or overactivation of autophagy machinery leads to autophagic neuronal cell death. Mitochondrial dysfunction or deficient mitophagy can promote the development of neurological diseases, such as ischemic cerebrovascular pathologies, AD, and PD [176, 178].

#### Autophagy and BBB

Several studies have indicated that the disruption of the BBB is a crucial biological event in the development and progression of various neurological disorders [179]. Certain factors, such as hypoxia and oxidative stress, stimulate autophagy in brain ECs, thereby influencing the BBB integrity [179, 180]. The disruption of the BBB under ischemic conditions is closely associated with the loss of TJs and BM [181]. The accumulation of H^+^ ions and cellular acidosis triggers ROS [182]. In response to ROS production, HDAC inhibitors promote autophagy and remove the damaged organelles to maintain the BBB integrity. The levels of ZO-1 and claudin-5 are restored in ECs exposed to oxidative stress [183, 184]. The abnormal autophagy response degrades essential compartments of the BBB structure, such as endothelial TJs, leading to the removal of BBB integrity. The leakage of the BBB allows several substances to enter the brain parenchyma and promote inflammatory responses [185]. Generally, autophagy is a crucial mechanism for maintaining the BBB integrity; however, dysregulated autophagy promotes BBB breakdown and exacerbates brain injury.

#### Autophagy and neural tissue regeneration

Neural tissue regeneration is activated to restore the damaged neural tissue. Unlike other tissues, CNS has a limited ability to regenerate injured cells [186]. For neural regeneration, neural stem and progenitor cells at specific brain sites are recalled and stimulated to promote neurogenesis and tissue healing [187]. These cells undergo dedifferentiation, in which they rapidly proliferate and further commit to specific cell lineages [188]. Of note, differentiating cells are recruited to the injured sites to replace dead neurons. The phenomenon of cell recruitment and migration is controlled by chemical and molecular cues in gradient patterns. The migrated cells mature into particular cell types that are required for tissue regeneration [189]. It has been shown that autophagy can also control regenerative mechanisms related to the CNS and peripheral nervous system. Moreover, it shows the potential to promote neural regeneration and facilitate functional recovery after brain injury [190, 191].

#### Autophagy, mitophagy, and apoptosis

Hyper- and hypoactivation of autophagy and its consequences on apoptosis are significantly involved in neurodegenerative processes after ischemic stroke [192]. As previously discussed, both intrinsic (mitochondria-mediated) and extrinsic (death receptor-mediated) pathways are engaged in ischemic stroke, with mitochondrial dysfunction serving as a pivotal contributor [54, 193]. The crosstalk between autophagy and apoptosis in ischemic stroke has garnered attention, as dysregulated autophagy leads to excessive apoptosis, which exacerbates ischemic injury and leads to neurodegeneration [194]. This interplay mainly drives mTOR, a major regulator of autophagy [193]; Beclin-1, a molecular switch between autophagy and apoptosis controlled by Bcl-2 family proteins [195]; and p53 [196], a central regulator of stress responses that plays a dual role [197, 198].

Ischemia may trigger AD pathogenesis by disrupting autophagy, mitophagy, and apoptosis, as reflected in the expression of *BECN1*, *BNIP3*, and *CASP3* genes, respectively [192]. To elucidate the crosstalk between autophagy and apoptosis, researchers aimed to explore whether and how the aforementioned genes are involved in the neuronal death post-ischemia in CA1 [199], CA3 [200], and medial temporal lobe cortex [201], and whether their expression varies across brain regions and time points [192]. To this end, utilizing quantitative PCR, authors analyzed the gene expression in a rat model of ischemic AD following 10-minute transient global ischemia, with survival times of 2, 7, and 30 days, and within a 2-year follow-up. Research on CA3 region with survival times of 2, 7, and 30 days showed persistent downregulation of *BNIP3* (mitophagy), delayed upregulation of *BECN1* (autophagy), and a biphasic pattern for *CASP3* (apoptosis) being downregulated at day 2 but upregulated from days 7 to 30. As there were no involvement of the *BNIP3* along with the *CASP3* in this region, it may be concluded that although neurons initially combat apoptosis, they eventually fail due to mitochondrial dysfunction and autophagy failure [200]. Moreover, research on CA3 region within a 2-year follow-up period demonstrated decreased expression of *BECN1* and *BNIP3* with upregulation of *CASP3* over a period of 0.5 year. Then, during 1–2 years, *BNIP3* and *CASP3* significantly increased, while *BECN1* remained variable. These imply that although autophagy is unable to efficiently remove damaged proteins and organelles in the long term, mitochondrial dysfunction endures for months after ischemia, and neurons attempt to compensate this through eliminating damaged mitochondria [192].

In the CA1 region, researchers observed that *BECN1* was not significantly altered within 30 days post-ischemia. Nevertheless, *BNIP3* and *CASP3* were upregulated on day 2, but returned to baseline on days 7 and 30. The findings suggest that mitophagy and apoptosis accompany dramatically in delayed neuronal death in CA1 post-ischemia. The early involvement of apoptosis, along with lack of autophagic support, explains the high susceptibility of CA1 neurons to ischemic injury [199]. Morover, research on medial temporal lobe has yielded significant results. The atrophy of this region has proven to be associated with higher risks of memory impairment and is significantly affected by disease pathogenesis [202]. Research on this region has shown early upregulation of *BECN1* and delayed upregulation of *CASP3* and *BNIP 3*. These results illustrate that autophagy initially serves as a protective mechanism, and may delay neurodegeneration in this region. However, the autophagic protection does not endure chronically, and neurons progressively undergo cell death [201]. In conclusion, while autophagy may offer early protection, its inability to maintain neuronal homeostasis, together with delayed mitophagy and extended apoptotic activation, all contribute to ischemia-induced neurodegeneration [199–201]. The long-term CA3 study further supports this [192].

#### Autophagy as a therapeutic approach

Autophagy regulators may be beneficial in preventing the progression of ischemic-stroke injuries [203]. It has been proposed that selective inhibition of autophagy following ischemic stroke, particularly in the pre-hospital phase, could provide neuroprotection before initiating definitive treatment in hospitalized patients [204]. Thus far, a wide range of autophagy modulators has been studied for the treatment of ischemic stroke [205]. For instance, the neuroprotective roles of Lithium, Metformin, and Rapamycin have beem proposed [206–208]. Moreover, Sevoflurane, an FDA-approved anesthesia drug, has demonstrated neuroprotective properties against ischemia by inhibiting autophagy hyperactivation through the PTEN/AKT1/mTORC1 signaling [209]. Additionally, it has been shown that downregulation of miR-338-3p following ischemic injury can minimize neuronal injury by activating Akt/mTOR signaling and decreasing autophagy [210]. In a rat model of ischemic stroke, long non-coding RNAs (lncRNAs) were shown to promote cerebral protection during ischemic postconditioning by suppressing excessive autophagy [211]. In spite of this, controversies persist regarding the application of these regulators in the clinical setting, indicating a lack of understanding of their underlying mechanisms and connections [205] (Table 1).

**Table 1:** Effect of treatment with autophagy regulators on animal models of ischemic stroke

Animal model	Treatment	Treatment Protocol	Key results	REF.
Rat model of MCA occlusion/reperfusion (MCAO/R)	Rapamycin	Macrophage-conditioned medium (MCM) and fibroblast pre-treated (20 nM for 24 h)	-regulated fibrosis -suppressed the migration and differentiation of fibroblasts and Col1 synthesis	[212]
Mouse model of MCA occlusion	Phosphodiesterase 10 A (PDE10A) TAK-063	Orally administered in the post-acute stroke phase, starting 5 days post-stroke (0.3 mg/kg or 3 mg/kg)	-improved cerebral microcirculation -enhanced neurological function -reduced infarct volume -increased neuronal survival -reduced brain edema -increased blood-brain barrier integrity	[213]
Mouse OGD/R model	AZD2014	Primary neuronal cultures from Wistar rat embryos treated either during OGD, 24 h before OGD, or for 24 h following OGD	-promoted cell death -reduced hamartin, a known neuroprotective mediator	[214]
Rat model of cerebral ischemia/reperfusion (CIR)	Minocycline	Intragastric administartion (50 mg/kg)	-upregulated levels of SYP and PSD-95 -p62 was downregulated in the hippocampus -LC3-II abundance and the LC3-II/I ratio were upregulated in the hippocampus	[215]
Rat model of cerebral ischemia/reperfusion (CIR)	Rapamycin	injected via the tail vein at 0.5 h before MCAO/R(150 µg/k)	-reduced Evans blue extravasation and brain water content -promoted the distribution of ZO-1 on cell membranes -reversed the decreased level of tight junction protein zonula occludens-1 (ZO-1)	[216]
Mouse OGD/R model	Astrocytic N-myc downstream-regulated gene 2 (NDRG2)	cells were infected using lentivirus, and infected cell lines were screened by puromycin	-promoted neuronal apoptosis and autophagy by dephosphorylating and blocking the PI3K/Akt/mTOR signaling	[217]
Mouse model of MCA occlusion/reperfusion (MCAO/R)	Astrocyte-derived exosomal nicotinamide phosphoribosyltransferase (Nampt)	transfected into primary astrocytes or neurons using Lipofectamine	-ameliorated neuronal injury by targeting AMPK/mTOR signaling -increased autophagy in hypoxic neurons -decreased cell death	[218]
Rat model of cerebral ischemia/reperfusion (CIR)	Rapamycin	intraperitoneal injection (250 µg/kg for 24 h)	-infarct areas were reduced -motor defects improved - attenuated cellular apoptosis -recovered mitochondrial function	[219]

This table summarizes the outcomes of various studies investigating the impact of autophagy-regulating treatments on animal models subjected to ischemic stroke

## Conclusion

Autophagy plays a central role in the aberrant and neurotoxic proteins clearance and maintenance of cellular homeostasis following ischemic stroke. This complicated phenomenon, through cell protection, inflammatory suppression, oxidative stress regression, and creation of a desirable microenvironment, could improve cell survival, axonal regeneration, neural tissue repair, brain function, and homeostasis [190]. Although autophagy facilitates neuroprotection and repair of neural tissue, excessive or dysregulated autophagy may cause neuronal damage [192]. Despite increasing insights, this topic remains poorly explored. Further research is required to elucidate the precise mechanisms underlying region-specific and time-dependent autophagic responses in different brain areas. Directed focus on developing region-specific, time-sensitive interventions that promote autophagy and mitophagy without excessive apoptosis is recommended for future research. Identification of pharmacological agents or gene-based therapies for such purposes may be beneficial. To optimize therapeutic approaches, additional research is needed to identify or develop biomarkers to monitor autophagy dynamics in clinical settings.

## Data Availability

No datasets were generated or analysed during the current study.

## Abbreviations

AGEs: Advanced Glycation Endproducts
AD: Alzheimer’s Disease
ATGs: Autophagy-related Genes
BBB: Blood-Brain Barrier
BECN1: Beclin-1
BM: Basement Membrane
BNIP3: BCL2/adenovirus E1B 19 kDa protein-interacting protein 3
CBF: Cerebral Blood Flow
CNS: Central Nervous System
CSF: Cerebrospinal Fluid
DA: Dopamine
ECM: Extracellular Matrix
ECs: Endothelial Cells
HIF-1α: Hypoxia-inducible factor 1-alpha
IL: Interleukin
ISF: Interstitial Fluid
JAM: Junctional Adhesion Molecule
LC3: Microtubule-associated proteins 1 A/1B light chain 3B
LRP1: Low-density lipoprotein receptor-related protein 1
MAPK: Mitogen-activated protein kinase
MHC: Major Histocompatibility Complex
MMPs: Matrix Metalloproteinases
mTOR: Mechanistic Target of Rapamycin
NF-κB: Nuclear Factor kappa-light-chain-enhancer of activated B cells
NRF2: Nuclear Factor Erythroid 2-Related Factor 2
PD: Parkinson’s Disease
PDGF: Platelet-derived growth factor
PI3K: Phosphoinositide 3-kinase
PINK1: PTEN-induced kinase 1
ROS: Reactive Oxygen Species
Shh: Sonic Hedgehog
SQSTM1: Sequestosome-1
TGF-β: Transforming Growth Factor Beta
TFEB: Transcription Factor EB
TJ: Tight Junction
TNF-α: Tumor Necrosis Factor Alpha
UPS: Ubiquitin-Proteasome System
ZO-1: Zonula Occludens-1
